# Bilateral Anterior Shoulder Dislocations Following a Clozapine-Induced Seizure

**DOI:** 10.7759/cureus.45778

**Published:** 2023-09-22

**Authors:** Lewis Hong

**Affiliations:** 1 Intensive Care Unit, Royal Perth Hospital, Perth, AUS

**Keywords:** seizure triggers, therapeutic drug monitoring, clozapine side effects, bilateral anterior shoulder dislocation, risk of seizure

## Abstract

Posterior shoulder dislocations are a recognised complication of generalised seizure episodes. Although less frequent, anterior shoulder dislocations are now being acknowledged as an emerging consequence. Particularly when they occur bilaterally, they can contribute to diagnosing a seizure disorder in a patient who shows no other signs during the post-ictal period. This article presents a case of bilateral anterior shoulder dislocations in an otherwise physically healthy young Sudanese gentleman following a generalised seizure episode on clozapine for a schizoaffective disorder.

The case aims to raise awareness of the occurrence of this phenomenon and emphasises the importance of timely diagnostic testing, seizure prophylaxis, and follow-up to minimise the risk of further seizure episodes and potential consequences. Additionally, there is a discussion regarding the utility of monitoring clozapine concentrations.

## Introduction

Seizures represent a potentially devastating life event, carrying the risk of both physical and mental harm and exerting a detrimental influence on a patient's social well-being [1]. Diagnosing seizures can pose a challenge due to their diverse presentations and the potential overlap with conditions like syncope or encephalopathy, which may share similar symptoms such as confusion and tonic-clonic movements. This difficulty is exacerbated when seizures go unwitnessed, requiring clinicians to rely on indicators like post-ictal confusion, incontinence, and lateral tongue biting as key signs of a generalised seizure event [1]. Accurate and timely diagnosis is important, as treatment is readily available and the consequences of uncontrolled seizures can be devastating and lifelong.

Shoulder dislocation following a generalised seizure is a well-known phenomenon and one of the clues suggested to clinicians that a seizure event may have occurred, with an incidence of around 0.6% [2]. Commonly, the dislocation is posterior and can be either unilateral or bilateral. It is, however, becoming more recognised that anterior dislocations can also occur [3,4].

While having traditionally been associated with primary epilepsy due to prevalence, seizure-related shoulder dislocations can arise from any cause of generalised seizure, such as hypoglycemia, drug and alcohol withdrawal, and adverse effects of prescription medication [5-7]. Clozapine, a potent antipsychotic valued for its effectiveness in treating schizophrenia and schizoaffective disorder, is one medication known to have the potential to induce seizures as an adverse effect [7]. A comprehensive work-up is required to ascertain potential precipitants of seizures, as the long-term management of epileptic seizures is vastly different from non-epileptic seizures [1].

This case report adds another instance of bilateral anterior shoulder dislocations following a seizure to the literature and intends to highlight the importance of comprehensive seizure work-up in patients who present with this condition with a vague history.

## Case presentation

A 24-year-old gentleman of Sudanese origin was admitted to a psychiatry ward in a rehabilitation facility following a relapse of treatment-resistant schizoaffective disorder. His background medical issues included a vague history of a possible myocardial event and a possible seizure episode in 2018; however, follow-up magnetic resonance imaging (MRI) did not reveal any structural brain lesions likely to cause seizures, and an electroencephalogram did not reveal any focal or generalised epileptiform discharges. The patient was also found to be negative for voltage-gated potassium channel complex antibodies and anti-N-methyl-D-aspartate (NMDA) antibodies; thus, anti-convulsant medications were subsequently not commenced.

On the morning of his 57th day of admission, he was noted to have woken up with severe bilateral shoulder pain, having been well the night before, albeit inebriated from drinking a bottle of red wine during a period of leave from the ward. On examination, there was a marked reduction in passive and active shoulder flexion and abduction bilaterally, with movements to approximately 90 degrees before restrictions due to severe pain and mechanical resistance. No neurovascular deficits were identified, and no other symptoms were noted. All vitals were within normal limits, his blood glucose level was 5.2 mmol, and a urine drug screen was negative. His medications at the time were 500 mg clozapine nocte, 5 mg olanzapine nocte, 100 mg aspirin mane, and regular docusate, senna, and macrogol for constipation.

**Figure 1:**
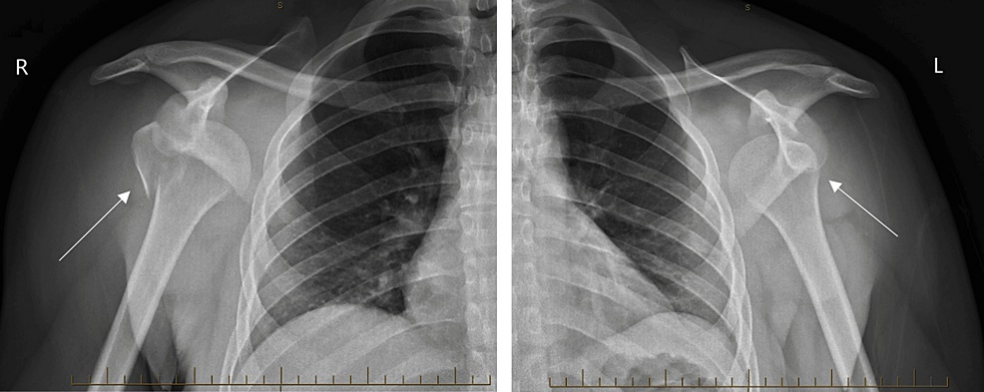
Bilateral shoulder X-rays showing bilateral anterior dislocations. There is also a fracture through the greater tuberosity of the right humerus.

Initial X-rays on the morning of the incident showed bilateral anterior shoulder dislocations with a fracture through the greater tuberosity on the right and a possible Bankart lesion on the left (Figure 1). A full blood count, urea and electrolytes, calcium, magnesium, and phosphate were remarkable for a slightly raised white cell count of 15.8 (neutrophils 13.74), with all other results being within normal limits. C-reactive protein and troponin, done as part of the institution's clozapine monitoring policy, were also within normal limits.

The patient was commenced on 100 mg tapentadol slow release for pain and transferred to a tertiary hospital, where both shoulders were reduced in the emergency department under sedation via the Kocher technique. Post-reduction X-rays subsequently showed a Hill-Sachs lesion on the left shoulder. A computer tomography (CT) head scan was performed to query potential causes of seizures; however, this returned unremarkable results. No EEG or further work-up was done, and no anti-convulsant medications were started. The patient was placed in bilateral broad arm slings and transferred back to the psychiatric ward.

While a seizure was thought to be a potential precipitating factor, at this point there had not been any seizure activity witnessed or any post-ictal signs noted, such as incontinence, confusion, or lateral tongue biting. Furthermore, bilateral anterior shoulder dislocations were atypical for a seizure, and a CT head was unremarkable. Therefore, no further seizure investigations (e.g., MRI or EEG) were done at this point.

**Figure 2:**
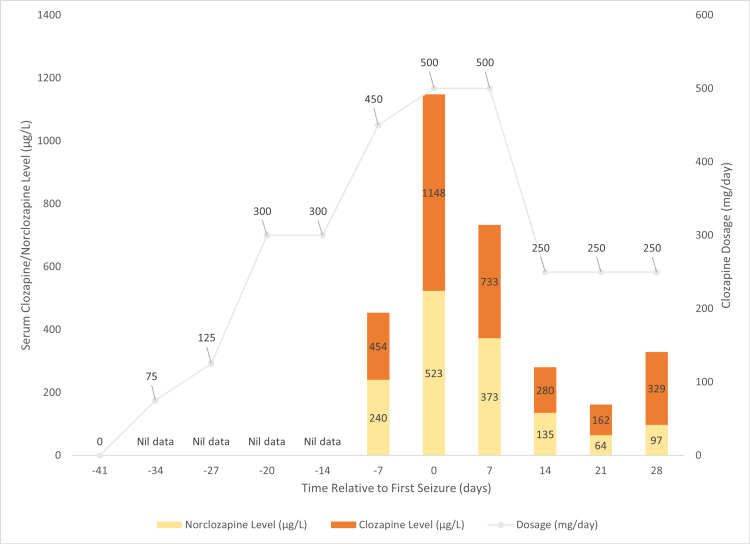
Clozapine dosage (mg), clozapine levels (µg/L), and norclozapine levels (µg/L) timed relative to the first seizure (t=0). The second seizure occurred at t=7. The clozapine to norclozapine ratio is ≥2 during the time when the seizures occurred. *Signifies a seizure event.

Seven days later, a generalised tonic-clonic seizure was witnessed. A repeat CT head was once again unremarkable; however, an EEG confirmed epileptiform waves. Further work-up revealed that the patient had a clozapine concentration of 1148 μg/L and a norclozapine concentration of 523 μg/L. The patient had been started on clozapine earlier in the admission and up-titrated to a dose of 500 mg nocte (Figure 2).

Upon identifying the elevated serum concentration and given concerns of a clozapine-induced seizure, the patient's clozapine was initially withheld, then subsequently restarted at a lower dose of 250 mg nocte. He was also started on 500 mg of sodium valproate twice a day. The clozapine concentration subsequently dropped to subtherapeutic levels in a span of six days and has remained that way since. Given the stability of the patient’s psychiatric presentation and the risk of further toxicity, the decision was made to maintain the lower dose despite the subtherapeutic concentration.

Follow-up X-rays one week later showed that the greater tuberosity of the right humerus remained displaced. The patient subsequently underwent an open reduction internal fixation of the right shoulder with proximal humerus internal locking system (PHILOS) plating and rotator cuff repair 24 days after the initial injury. No further seizure activity was noted during the remainder of his admission, and the patient appeared to tolerate the sodium valproate without issue. He remained psychiatrically stable and was discharged to his usual residence after 115 days from admission.

## Discussion

Bilateral anterior shoulder dislocations are more commonly seen following trauma and electric shocks due to the nature of requiring an abducting, extending, and external rotating force for the dislocation to occur. The movements during seizures predispose more towards posterior shoulder dislocation, as the more powerful internal rotators (latissimus dorsi, pectoralis major, and subscapularis) overpower the weaker external rotators of the humerus (infraspinatus and teres minor), causing adduction and internal rotation strong enough to dislocate the humeral head posteriorly [5].

Despite this, bilateral anterior shoulder dislocations following a seizure are a well-documented phenomenon. In 2013, Ballesteros et al. described 44 cases in the literature of bilateral anterior shoulder dislocations following seizures [2], and further cases have been described since then [3,8]. While some cases had pre-disposing factors of prior dislocations or shoulder injuries, other cases described dislocations in the previously uninjured and anatomically typical shoulder, suggesting that there is the possibility of this type of injury in any person who has a generalised seizure.

Initial investigations into seizures consist of blood tests and neuroimaging. CT is a widely available and quick method of neuroimaging. However, in terms of seizures, it is found to only be useful when there is evidence of an abnormal neurological examination or a history of head injury, fever, immunosuppression, malignancy, or focal motor or sensory onset of seizure [9,10]. MRI is the imaging of choice due to its ability to detect small lesions; however, it is less readily available [11].

In the above case, given the initial diagnostic ambiguity regarding the cause of the bilateral anterior shoulder dislocation, an EEG on the first transfer may have identified the underlying seizure disorder earlier. If performed within 24-48 hours of a first seizure, the EEG shows substantial abnormalities in approximately 70% of cases [12]. Additionally, a recent case series found that bilateral shoulder dislocations, encompassing both anterior and posterior dislocations, were frequently linked to a first generalised seizure episode [13].

Goudie et al. have proposed a classification for seizure-related shoulder instability, comprising a spectrum of disorders within which it is possible to identify four broadly distinct clinical entities: (1) first-time dislocation; (2) recurrent instability; (3) chronic locked dislocation; and (4) fracture dislocation. The purpose of this classification was to highlight the increasing shoulder instability with each episode of seizure [14]. This case is unique as it fits into both the first and fourth categories, conveying that some individuals may end up with significant instability after a single seizure-dislocation episode and highlighting the importance of timely treatment to prevent further shoulder instability.

Clozapine is a second-generation antipsychotic that exerts its effects through interaction with various neuroreceptors, including those for dopamine, serotonin, and muscarinic receptors [15]. It is associated with a spectrum of adverse effects, such as agranulocytosis, cardiac toxicity, and diabetes [15,16]. Notably, it can also lead to seizures, particularly when administered in higher doses [7]. Clozapine is metabolised by the liver enzymes CYP2D6, CYPP1A2, and CYP3A4 until these enzymes are fully saturated [17,18]. Thereafter, the serum concentration of clozapine rises rapidly. It is generally considered that a minimum trough level of 350 ng/mL of clozapine in plasma is associated with a favourable therapeutic response, with levels surpassing 600 ng/mL carrying an elevated risk of dose-dependent adverse effects [15].

While the active metabolite of clozapine, norclozapine, has questionable clinical effects, the clozapine-to-norclozapine ratio has been proposed as a tool to assess medication adherence, assist with dose titration, and minimize toxicity [16,19]. A norclozapine level that is equivalent to roughly half of the clozapine level has been suggested to be the optimal ratio (i.e., a clozapine to norclozapine ratio of two). If it is less than half (i.e., if the ratio is greater than two), this indicates that the sample was either non-trough or that the metabolism enzymes are fully saturated.

Although several reasons could be attributed to the sudden rise in serum concentration in our current patient, one possibility is that the 500 mg dosage exceeded the capacity of the CYP enzyme metabolism, resulting in an oversaturation and a sudden rise in concentration. This is supported by the clozapine-to-norclozapine ratio being greater than two during the time when the seizures occurred. However, clozapine metabolism is affected by various factors, including common lifestyle activities such as smoking and alcohol use [20], so identifying a single cause can be difficult.

The established approach for managing clozapine-induced seizures involves dose-reducing or discontinuing the medication. In cases where continuing clozapine at a higher dose is deemed necessary, sodium valproate is suggested as the preferred antiepileptic, with some evidence also supporting the use of lamotrigine, gabapentin, and topiramate [7,15]. Nevertheless, it is worth noting that well-designed clinical trials specifically addressing clozapine seizure prevention are currently lacking [7]. In this instance, the patient's treatment plan involved initiating a regimen of 500 mg of sodium valproate twice daily, coupled with a reduction in the dosage of clozapine. This adjustment appeared to successfully prevent further occurrences of generalised seizures.

## Conclusions

The presentation of bilateral anterior shoulder dislocation, particularly when accompanied by a vague medical history, should prompt careful consideration of seizure as a potential underlying cause. Moreover, shoulder dislocations can occur following seizures, even in those with no prior history of joint or connective tissue disorders. To ensure a timely and accurate diagnosis, an acute EEG performed within the initial 24-48 hours following a suspected seizure can provide valuable insight, enabling the prompt initiation of appropriate seizure prophylaxis. Clozapine is an antipsychotic well known to cause seizures at higher doses, especially if plasma levels surpass 600 ng/mL. When managing patients undergoing clozapine maintenance therapy, maintaining a clozapine-to-norclozapine ratio of two emerges as the optimal target. A higher ratio might indicate potential oversaturation of metabolic enzymes, leading to an increased risk of adverse effects.
